# 间质病样肺血管内大B细胞淋巴瘤的临床病理及基因分析

**DOI:** 10.3760/cma.j.cn121090-20240424-00160

**Published:** 2024-09

**Authors:** 宏艳 刘, 诗璇 刘, 晓伟 王, 蓓 王, 秀红 王, 芳 于, 振玲 李, 定荣 钟

**Affiliations:** 1 中日友好医院病理科，北京 100029 Department of Pathology, ChinaJapan Friendship Hospital, Beijing 100029, China; 2 中日友好医院血液科，北京 100029 Department of Hematology, ChinaJapan Friendship Hospital, Beijing 100029, China

**Keywords:** 淋巴瘤，B细胞, 间质性肺病, 基因突变, Lymphoma, B-cell, Lung disease interstitial, Gene mutation

## Abstract

**目的:**

探讨肺血管内大B细胞淋巴瘤的临床病理及基因突变特征。

**方法:**

回顾性分析中日友好医院2018年4月至2023年5月病理确诊的8例肺血管内大B细胞淋巴瘤患者的临床及病理资料，二代测序（NGS）方法检测其中6例基因突变情况，并对患者进行随访。

**结果:**

患者男1例，女7例，中位年龄64（45～66）岁，临床症状以呼吸系统症状最常见（7例），B症状2例，继发噬血细胞综合征2例；胸部高分辨率CT均显示双肺多发弥漫磨玻璃影，肺功能检查轻至中度通气或弥散功能障碍6例，血气分析示低氧血症2例，伴Ⅰ型呼吸衰竭2例；血清LDH升高（7/8），β_2_微球蛋白升高（2/8），神经元特异性烯醇化酶升高（7/8），外周血淋巴细胞总数减少（7/8），临床分期均为Ⅳ期。组织学特征：肿瘤细胞充满肺泡间隔的小静脉和毛细血管，肿瘤细胞体积大，核仁明显，核分裂象常见，8例均可见肿瘤细胞浸润血管外周围肺组织。免疫表型：8例肿瘤细胞均弥漫表达B细胞抗原CD20、CD79a，血管内皮标志CD31和CD34染色显示肿瘤细胞位于血管内、血管壁及血管周围；1例为生发中心型，7例为非生发中心型，2例为双表达淋巴瘤，所有病例EB病毒编码的小RNA（EBER）均阴性。NGS检测突变频率前5位的基因分别为MYD88（5/6）、PIM1（5/6）、CD79B（4/6）、TCF3（4/6）、TP53（3/6）。8例中的7例接受R-CHOP方案为基础的化疗，6例化疗后完全缓解，1例死亡，1例失访。

**结论:**

肺血管内大B细胞淋巴瘤罕见，影像学表现与间质性肺病鉴别困难，经支气管镜肺活检是确诊的有效方法，基于分子分型联合布鲁顿酪氨酸酶抑制剂的免疫化疗未来可以让患者生存获益。

## Abstract (en)

**Objective:**

To investigate the clinicopathological features and genetic mutation status of pulmonary intravascular large B cell lymphoma.

**Methods:**

The clinicopathological data of eight patients diagnosed with pulmonary intravascular large B cell lymphoma, from April 2018 to May 2023, were retrospectively analyzed. The genetic profile of six patients was detected via next-generation sequencing（NGS）and followed up.

**Results:**

All patients included one male and seven females, with a median age of 64 years（ranging from 45 to 66 years）. Respiratory symptoms were the most common（7 cases）, B symptoms in two cases, hemophagocytic syndrome in two cases. Multiple diffuse ground-glass opacities in both lungs were observed based on the high-resolution chest CT scan. Six cases of mild to moderate ventilation or diffusion dysfunction were observed based on the pulmonary function tests. Moreover, two cases of hypoxemia and two cases with type Ⅰ respiratory failure were recorded. The serum lactate dehydrogenase level increased（7/8）, β2-MG level increased（2/8）, neuron-specific enolase level increased（7/8）, total number of peripheral blood lymphocytes decreased（7/8）, and clinical stages were all stage Ⅳ. The neoplastic lymphoid cells were lodged in the lumina of venules and capillaries of the alveolar septum; the tumor cells were large, with prominent nucleoli and frequent mitotic figures. The malignant cells were detected in the extravascular surrounding lung tissue in all cases. The tumor cells expressed mature B cell-associated antigens CD20 and CD79a, and the vascular endothelial markers CD31 and CD34 showed that the tumor cells were filled in the blood vessels, infiltrated blood vessel walls, and perivascular areas. One case was germinal center-type, seven cases were non-germinal center-type, two cases were double-expressing lymphoma, and all cases were EBER-negative. Furthermore, the top five genes with mutation frequencies detected by NGS were MYD88（5/6）, PIM1（5/6）, CD79B（4/6）, TCF3（4/6）, and TP53（3/6）. Of the eight cases, seven patients received R-CHOP-based chemotherapy, six cases had complete remission after chemotherapy, one case died, and one case was lost to follow-up.

**Conclusion:**

Pulmonary vascular large B cell lymphoma is rare, which shares similar patterns with interstitial lung disease on imaging. Transbronchial lung biopsy is an effective method to confirm the diagnosis. Immunochemotherapy with BTK inhibitors can provide a survival advantage for patients in the future based on molecular typing.

原发性肺B细胞淋巴瘤（primary pulmonary B-cell lymphoma，PP-BCL）是一种罕见疾病，在非霍奇金淋巴瘤中占比<1％，占结外非霍奇金淋巴瘤的3％～4％。常见亚型包括肺黏膜相关淋巴组织结外边缘区B细胞淋巴瘤、肺原发弥漫大B细胞淋巴瘤（DLBCL）和肺淋巴瘤样肉芽肿，罕见类型包括原发性渗出性B细胞淋巴瘤和血管内大B细胞淋巴瘤（intravascular large B-cell lymphoma，IVLBCL）[1]–[2]。IVLBCL过去被描述为恶性血管内皮瘤病，是一种罕见大B细胞淋巴瘤的结外亚型，通常累及小血管，特别是毛细血管和毛细血管后微静脉，通常不累及淋巴管。《血管内大B细胞淋巴瘤诊治中国专家共识（2023年版）》[3]根据临床表现将IVLBCL分为3个亚型：经典亚型、皮肤亚型和噬血细胞相关亚型，西方国家常见经典亚型，以皮肤及中枢神经系统受累为主，亚洲国家常见噬血细胞相关亚型，伴有多系统受累、肝脾肿大和全血细胞减少。然而，原发于肺部的IVLBCL通常起病隐匿，呼吸系统症状和胸部放射学表现不典型或非特异性[4]，临床诊断非常困难，目前国内外对此仅有少数个案报道，因此我们对本中心8例肺IVLBCL患者的临床、病理资料及基因突变特征进行系统性回顾分析，为临床提供肺IVLBCL的诊疗经验。

## 病例与方法

1. 病例资料：研究对象为中日友好医院2018年4月至2023年5月期间，在中日友好医院确诊且临床资料完整的8例IVLBCL患者，所有病例均由2名高年资病理医师按照世界卫生组织2017年修订的《淋巴造血系统肿瘤分类》独立作出诊断，标本均为经支气管镜肺活检或经支气管冷冻肺活检获得。噬血细胞综合征根据2004年噬血细胞性淋巴组织增生症诊断标准[5]诊断。收集患者住院期间的实验室检查、影像学资料和治疗随访信息。

2. 组织学观察、免疫组织化学染色及EB病毒原位杂交：所有组织标本使用3.7％中性甲醛液固定24 h，常规石蜡包埋，4 µm连续制片，苏木精-伊红（HE）染色，光镜观察。免疫组化采用EnVision二步法，二氨基联苯胺（DAB）显色，一抗分别为CD20、CD79α、CD10、BCL-6、MUM-1、BCL-2、CD3、CD5、CD31、CD34、ERG、c-Myc、Ki-67、P53、TTF-1。EB病毒原位杂交应用EB病毒编码的小RNA（EBER）检测试剂盒，操作步骤按照试剂说明书进行，设立相应的阳性对照。EnVision试剂盒、一抗、EBER检测试剂盒及探针均购自北京中杉金桥生物技术有限公司，二抗及显色剂为罗氏诊断产品（上海）有限公司试剂。

3. 基因测序：应用二代测序（NGS）技术检测肿瘤热点基因突变情况。使用QIAamp DNA FFPE组织试剂盒（QIAgen）从IVLBCL患者福尔马林固定石蜡包埋的肿瘤组织样本中提取基因组DNA，苏州吉因加生物医学工程有限公司人淋巴瘤基因突变检测试剂盒，BGI测序平台（华大基因）进行文库制备及联合探针锚定聚合测序法进行测序，基因Pannel包括413个淋巴瘤密切相关的基因蛋白编码区域的点突变、短片段的插入缺失突变、基因重排和融合变异，每个样本捕获目标区域平均有效深度≥500×。本研究对6例患者石蜡样本进行了NGS检测，将基因突变频率>10％定义为高频突变基因。

4. 治疗和随访：7例患者接受R-CHOP（利妥昔单抗+环磷酰胺+阿霉素+长春新碱+泼尼松）方案为基础的化疗4～6个周期，例2联合来那度胺口服，例6联合来那度胺和泽布替尼治疗，1例死亡，1例失访。通过门诊及电话联系方式进行随访，截止日期为2024年3月13日。

5. 统计学处理：本研究中的计量资料应用*M*（范围）进行统计描述，计数资料采用绝对数、构成比和百分率进行统计学描述。

## 结果

1. 临床特征：如表1所示，8例患者中，男1例，女7例，中位年龄64（45～66）岁。所有患者发病部位均为肺，未见皮肤及中枢神经系统累及，7例症状表现为胸闷、气短、活动后呼吸困难及间断发热，例5无明显症状仅体检发现肺部阴影，2例（例2、7）出现B症状，3例（例6、7、8）伴肝脾肿大，2例（例3、6）出现噬血细胞综合征；胸部CT均显示双肺多发弥漫磨玻璃影和结节影（图1A），呈间质性肺炎改变。6例行肺功能检查提示轻至中度阻塞性通气功能障碍及不同程度弥散功能障碍，2例（例1、4）血气分析提示低氧血症，例4和例8伴Ⅰ型呼吸衰竭；实验室检查：8例患者中，7例血清LDH升高；2例β_2_微球蛋白升高；7例神经元特异性烯醇化酶（NSE）升高；7例外周血淋巴细胞总数减少；临床分期均为Ann Arbor Ⅳ期。

**表1:** 8例肺血管内大B细胞淋巴瘤患者的临床特征

例号	年龄（岁）	性别	IPI评分（分）	LDH（IU/L）	β_2_-MG（mg/L）	NSE（ng/ml）	外周血淋巴细胞总数（×10^9^/L）	细胞起源分型	双表达淋巴瘤	治疗方案	治疗反应
1	45	女	1	214	2.10	23	0.84	GCB	NA	NA	NA
2	66	女	3	923	1.51	27	0.64	non-GCB	否	R-CHOP、来那度胺	CR
3	51	女	3	770	2.49	27	0.65	non-GCB	否	R-CHOP	死亡
4	64	女	4	1 377	5.24	44	0.37	non-GCB	否	R-CHOP	CR
5	66	女	3	308	2.27	13	2.26	non-GCB	否	R-CHOP	CR
6	62	女	5	975	11.60	25	0.81	non-GCB	是	R-CHOP、泽布替尼+来那度胺	CR
7	53	男	3	865	NA	79	0.50	non-GCB	否	R-CHOP	CR
8	56	女	3	1 689	NA	63	1.01	non-GCB	是	R-CHOP	CR

**注** β_2_-MG：β_2_微球蛋白；NSE：神经元特异性烯醇化酶；GCB：生发中心B细胞；non-GCB：非GCB；R-CHOP：利妥昔单抗+环磷酰胺+阿霉素+长春新碱+泼尼松；CR：完全缓解；NA：未获得

**图1:**
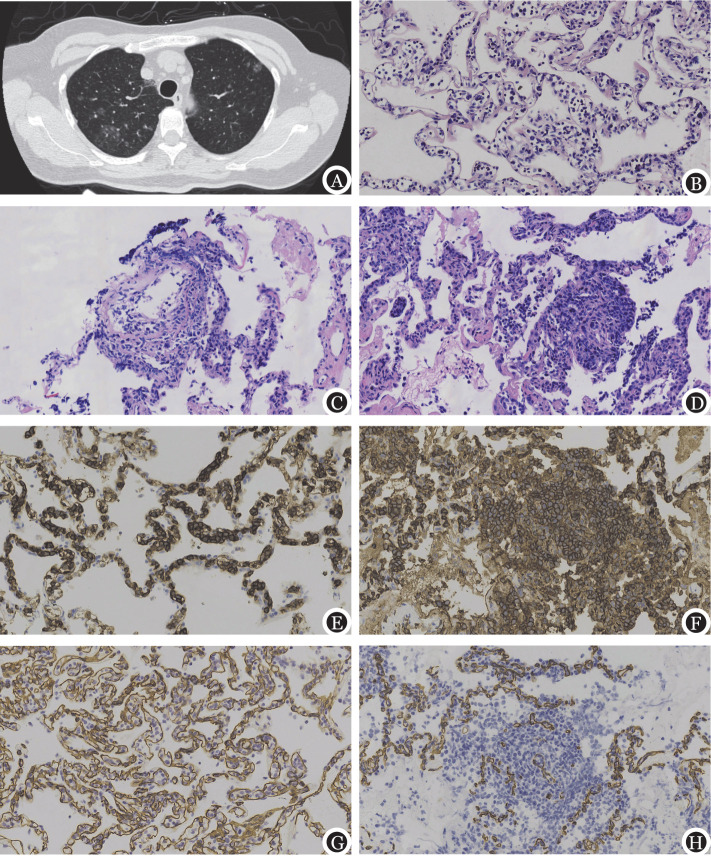
肺血管内大B细胞淋巴瘤影像学（例5）、HE染色及免疫组化（例3，×400） **A** 高分辨率CT显示双肺多发磨玻璃结节影；**B** HE染色显示肺泡间隔毛细血管腔间隙充满体积较大的异型的淋巴细胞；**C** 淋巴瘤细胞浸润血管壁；**D** 淋巴瘤浸润血管外周围肺组织，形成局限性微结节；**E、F** 免疫组化CD20染色显示肺泡间隔毛细血管腔内的异型淋巴细胞及CD20阳性的淋巴瘤细胞在血管外肺泡间隔形成微结节；**G、H** 免疫组化血管内皮标志CD34示毛细血管内及血管外的淋巴瘤细胞

2. 病理特征：8例活检肺组织镜下所见相似，肺泡间隔毛细血管及小静脉中可见体积较大的淋巴细胞单个或成簇分布，黏附于血管内皮或呈游离状态（图1B），所有患者均可见淋巴瘤细胞浸润血管外肺组织（图1C、D）；淋巴瘤细胞以大细胞为主，核圆形、类圆形或不规则，核膜常清晰，染色质较粗，核仁明显，核分裂象易见，形态类似中心母细胞或免疫母细胞。

所有患者免疫组化均表达B细胞抗原CD20（图1E、F）、CD79a，6例MUM1阳性，Ki-67增殖指数平均为80％，CD31和CD34血管内皮标志物可清晰显示血管内及周围的淋巴瘤细胞（图1G、H）。按照细胞来源（COO法则）分型，7例为非生发中心型（non-GCB），1例为生发中心型（GCB）；2例为c-Myc和BCL2双表达；EB病毒原位杂交均为阴性。

3. 基因突变特征：应用NGS技术检测6例IVLBCL患者，共检测到57个基因的116个突变位点，突变位点中位数为13（8～41）个，平均每例9.5个驱动突变，突变类型以错义突变最多见，其次是移码突变，突变频率前10位的基因依次为MYD88（5/6，83.3％）、PIM1（5/6，83.3％）、CD79B（4/6，66.7％）、TCF3（4/6，66.7％）、TP53（3/6，50.0％）、ETV6（2/6，33.3％）、CCND3（2/6，33.3％）、RELN（2/6，33.3％）、PCLO（2/6，33.3％），PRDM1（2/6，33.3％），所有鉴定出的MYD88突变都发生在热点p.L265P中，而所有CD79B突变位于ITAM结构域p.Y196的热点；例5检测到NOTCH2突变，例6检测到NOTCH1突变，例1检测到TET2突变。

4. 疗效和随访：8例患者，7例采用R-CHOP方案为基础的化疗，6例治疗后完全缓解，1例化疗第2天即死亡，1例转院后失访，中位随访时间为23（10～72）个月。

## 讨论

IVLBCL作为罕见侵袭性大B细胞淋巴瘤的亚型，起病即为晚期，IPI评分高，B症状以及神经系统和皮肤受累的发生率高[6]。以往文献报道IVLBCL肺部受累发生率为36％～50％[7]，本组8例患者为累及肺的经典型IVLBCL，老年女性为主，7例表现为非特异性的呼吸道症状，2例伴噬血细胞综合征。除与淋巴瘤相关的血清LDH和β_2_微球蛋白升高外，本组87.5％患者血清NSE升高及外周血淋巴细胞计数减少，研究表明NSE除了在实体肿瘤中升高，在DLBCL中有54.7％的阳性率，且non-GCB亚型DLBCL患者血清NSE平均值显著高于GCB亚型，血清NSE水平被认为是non-GCB亚型患者的独立预后因素[8]，因此在临床上难以鉴别肺病变性质时，可参照此肿瘤标志物的检测来协助诊断。此外，有研究报道IL-10在IVLBCL中的敏感度和特异度分别为80％和100％，可用于疗效监测[9]。IVLBCL累及肺的影像学表现与间质性肺病相似，PET-CT常提示肺弥漫磨玻璃结节及肝脾肿大伴代谢增高，虽无法明确诊断但对确定活检部位有一定帮助[10]。

IVLBCL累及肺的组织学表现为体积较大的淋巴细胞弥漫分布于肺间质血管中，特别是毛细血管，几乎所有患者可见肿瘤细胞浸润至血管壁及周围肺间质并局灶聚集形成微结节，部分肺泡腔内可见纤维素性渗出。淋巴瘤细胞增生所致的肺泡间隔增宽，可以解释高分辨率CT显示的肺透光度下降、肺间质样病变及弥漫性磨玻璃病变及结节[7]。患者肿瘤细胞的生长方式显示多样性，肿瘤细胞漂浮于血管腔，聚集填充和闭塞血管腔或黏附于血管内皮上生长，血管结构通常完整。肿瘤细胞被限定在血管腔内增殖，目前认为与肿瘤细胞缺乏CD29（整合素β-1）、CD54（ICAM1）黏附分子、参与跨血管迁移的各种趋化因子受体（CXCR5、CCR6、CCR7）以及基质金属蛋白酶q缺失或表达水平低相关[11]。

IVLBCL累及肺的鉴别诊断包括：①血管内NK/T细胞淋巴瘤，表达T细胞标志物及CD56，且与EB病毒感染密切相关[12]；②肺非肿瘤性病变（如间质性肺疾病），IVLBCL肿瘤细胞浸润血管壁外间质与炎症相鉴别，后者淋巴细胞较小，无异型性；也有报道粟粒性肺结核临床表现可类似IVLBCL[13]；③DLBCL累及血管及淋巴管，可在脉管内形成瘤栓，但同时伴有淋巴结肿大或结外脏器的占位性病变，需结合临床病史、影像学检查及组织病理学综合分析[3]；④淋巴瘤样肉芽肿，DLBCL的特殊亚型，90％以上发生于肺，是一种血管中心性和血管破坏性淋巴组织增生性疾病，EB病毒阳性的细胞同时表达CD20，常在肺内形成结节，可通过免疫组化血管内皮CD31和CD34染色鉴别IVLBCL；⑤转移性癌，成簇的癌细胞位于淋巴管或血管内，免疫组化染色广谱CK阳性而白细胞共同抗原LCA阴性。

常规细胞遗传学研究和患者来源的异种移植物的拷贝数阵列证实IVLBCL具有涉及1、6q、8p、9p、18、19q染色体和四倍体高度异质性复杂核型[14]–[15]。NGS分析结果显示IVLBCL为活化B细胞型DLBCL基因表达谱，MYD88和CD79B是其高频突变基因，分子分型归为MCD亚型或C5[16]–[17]。本组6例患者NGS结果显示MYD88、CD79B及PIM1是肺IVLBCL的高频突变基因，这与最近一项有关IVLBCL基因组图谱的研究相一致[18]，MYD88和CD79B是BCR/NF-κB信号通路突变基因，而PIM1在IVLBCL中的突变率为60％，是已知的体细胞超突变（SHM）靶标，PIM1调节NF-κB和JAK/STAT途径并促进原发性纵隔大B细胞淋巴瘤和经典霍奇金淋巴瘤的肿瘤免疫逃逸[19]–[20]。IVLBCL是否伴有噬血细胞综合征及是否存在肿瘤细胞血管外播散的组织学特征基因突变谱表达差异无统计学意义[18]。

除了皮肤亚型外，IVLBCL的侵袭性及非特异性表现常导致诊断延迟，从而预后较差，目前尚无最佳的治疗方案，早期给予利妥昔单抗的联合化疗可以使50％的患者达到完全缓解[2]。近年来布鲁顿酪氨酸酶（BTK）抑制剂在与MYD88激活突变相关的华氏巨球蛋白血症、原发性中枢神经系统淋巴瘤、慢性淋巴细胞白血病和边缘区淋巴瘤的治疗中取得了显著的疗效[21]，Wilson等[22]研究显示在MCD和N1亚型DLBCL中，接受伊布替尼联合R-CHOP方案治疗的年轻患者（年龄≤60岁）的3年无进展生存率为100％，而单独使用R-CHOP方案治疗的患者生存率分别为42.9％和50％。例6为老年女性患者，non-GCB型，NGS显示MYD88和CD79B突变，伴有噬血细胞综合征，临床分期ⅣB期，IPI评分为5分（高危），接受1个周期的R-CHOP方案化疗后因新冠感染停用利妥昔单抗，继而联合ZR（来那度胺和泽布替尼）-CHOP方案完成6个周期治疗后完全缓解，目前接受利妥昔单抗和泽布替尼维持治疗。BTK抑制剂对噬血细胞亚型IVLBCL疗效，证实了其在non-GCB型DLBCL治疗中的应用前景和价值。

作为单中心的回顾性研究，本文例数有限，随访时间偏短，个别患者失访或无法获得组织进行测序，难以通过生存分析获得影响预后的临床、病理和基因相关参数。但由于发病率极低，国内外有关肺IVLBCL的文献仅局限于个案报道，因此尚需积累病例资料进行大样本的临床研究或荟萃分析。

综上，发生于肺的IVLBCL是非常罕见的，临床及影像学表现具有迷惑性，经支气管镜肺活检对于确诊最有意义；基于分子遗传学特征的精准预后分层，期待联合BTK抑制剂的免疫化疗未来可以为IVLBCL患者带来更好的生存获益。
